# Navigating ortho care amidst war crisis: insights from Al-Aqsa Martyrs’ Hospital’s orthopedic department at Gaza Strip

**DOI:** 10.3389/fpubh.2025.1595477

**Published:** 2025-07-02

**Authors:** Abdulwhhab Abu Alamrain, Mohammed Halimy, Hitham Toman, Alaa Kassab, Majdi Alkhaldi, Yosef Alderdasawe, Mahmoud Abuaita, Ahmed Alzayyan, Mohammed Tahir, Ayman I. Harb

**Affiliations:** ^1^Faculty of Medicine, Al-Quds University, Jerusalem, Palestine; ^2^Orthopedic Department, Al-Aqsa Martyrs’ Hospital, Deir Al-Balah, Palestine; ^3^Faculty of Medicine, Islamic University of Gaza, Gaza City, Palestine; ^4^Faculty of Medicine, Ain Shams University, Cairo, Egypt; ^5^FAJR Scientific, Ann Arbor, MI, United States

**Keywords:** war, orthopedic, conflict, public health, preparedness & response, orthopedic trauma, resilience

## Abstract

The war in Gaza has triggered an unprecedented humanitarian crisis and severely impacted the healthcare system. It has overwhelmed orthopedic care at Al-Aqsa Martyrs’ Hospital, the only public hospital serving the middle region of the Gaza Strip. With over a million displaced individuals and a sharp rise in war-related injuries, the department has been operating under extreme constraints, including shortages of medical supplies, staff, and infrastructure. The orthopedic department, initially small and under-equipped, has rapidly expanded and adapted to handle mass casualties. This has involved restructuring staff shifts, implementing a three-team 24-h rotation system, and repurposing existing facilities to accommodate the influx of patients. Emergency care evolved around a fluidic triage system, and surgical capacity was extremely strained. The repurposed obstetrics and gynecology theaters and delivery rooms became primary operating rooms, with two-thirds of all surgeries being orthopedic-related. Innovative solutions, such as field hospitals and tents for dressing, have helped alleviate some of the pressure. The introduction of the Dressing Under General Anesthesia (DUGA) system has improved wound care for severe injuries. International collaboration, particularly with Médecins Sans Frontières (MSF) and other NGOs, has played a critical role in supplementing medical services and expertise, providing medical and surgical supplies, and facilitating certain procedures. Despite these adaptations, the department remains overwhelmed. Resource limitations have caused delays in essential procedures, leading to complications such as non-union fractures and infections. Additionally, coordination between local and international teams has been challenging, underscoring the need for a more structured response to improve efficiency. The study highlights the adaptation and resilience of the orthopedic department at Al-Aqsa Martyrs’ Hospital. It underscores the urgent need for policy reforms to enhance emergency preparedness, expand surgical capacity, and develop a sustainable, resilient healthcare system. Key recommendations include standardizing clinical protocols, strengthening supply chains, and supporting overburdened and burnout medical staff. Addressing these challenges is crucial to sustaining orthopedic care in Gaza’s war-torn healthcare system.

## 1 Introduction

The war in Gaza has triggered an unprecedented humanitarian crisis, severely impacting the region’s healthcare system (1, 2). According to the Palestinian Ministry of Health (MoH), the war has resulted in over 52,000 deaths and 119,000 injuries as of mid-May 2025 (3). Al-Aqsa Martyrs’ Hospital, the only public hospital serving the middle area of Gaza Strip, has become one of the epicenters of this struggle (4). Mass displacement from northern Gaza after evacuation orders, along with overcrowding and congestion of displaced people, have stretched the services provided beyond its limits (5). The surging of individuals, patients, and war-related injury numbers, compounded by bombardment and the closure of the Rafah border crossing, have overwhelmed its limited resources and infrastructure (2, 6, 7). Years of blockade and repeated conflicts had already weakened the healthcare system, putting it on the edge of collapse, with chronic shortages of medical supplies, equipment, and personnel (8, 9). The outbreak of the war exacerbated these challenges, pushing the hospital and orthopedic department to its limits. This department has expanded to address the unprecedented influx of patients and injuries, as limbs’ injuries were among the most common. This community case study explores how the orthopedic department at Al-Aqsa Martyrs’ Hospital adapted during the past year of conflict. By analyzing its response, the study sheds light on the broader struggles of delivering healthcare in war zones and emphasizes the critical need for building resilient healthcare systems to endure such crises.

## 2 Context

The hospital is a small facility built to serve fewer than 200,000 residents in the middle area, which has been forced to accommodate and serve over one million displaced individuals (10, 11). It consists of four buildings, two of which function as direct care facilities, while the others house the laboratory, blood bank, administrative department, IT department, and outpatient clinics. The two care buildings are as follows: one main building with three floors and a two-story building designated for obstetrics, gynecology, and neonatology. The total inpatient capacity was about 250. The orthopedic department did not have a designated inpatient section but rather a shared inpatient department with the general surgical inpatient department on the third floor of the main building.

Prior to the war, hospital and departmental preparedness for emergencies had been minimal. While some general response frameworks existed, there was no comprehensive plan that could adequately mitigate a prolonged crisis at this scale. The onset of mass casualties, compounded by mass displacement, exposed the structural fragility of the healthcare system. This prompted rapid, reactive, and ad hoc adaptations based on immediate needs and the on-the-ground situation.

Before the war, the hospital had limited surgical capacity, three general operating rooms, and two obstetrics and gynecology theaters. Reconstruction of the general surgical facilities began before the war but remains incomplete. With the beginning of the war, the obstetrics and gynecology department was moved to another private hospital, Al-Awda Hospital, under emergency response collaboration (12). The two-floor building was repurposed as a general inpatient building. The two functional operation theaters and the three previously delivery rooms and minor gynecology procedures were reformed into general theaters for different surgical departments, including orthopedic surgery.

**Figure 1:**
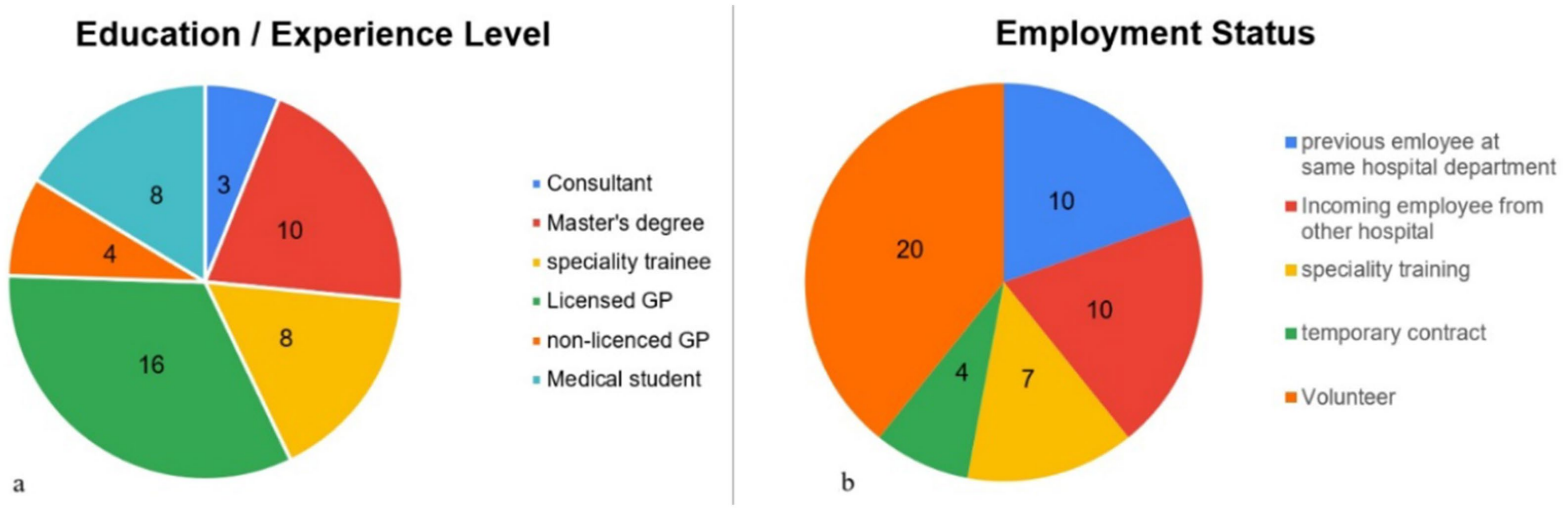
**(a)** Pie chart representing the education/experience level of orthopedic department staff as of November 2024. **(b)** Pie chart representing the employment status of orthopedic department staff as of November 2024.

Despite these limitations, Al-Aqsa Martyrs’ Hospital evolved into a critical trauma care hub. Enduring multiple bombings and evacuations, it was still striving to meet the demands of war-related injuries (4, 13–15). The orthopedic department, previously small and understaffed, has expanded rapidly with the support of volunteers, incoming displaced staff from other hospitals, newly recruited staff under emergency contract, and medical students (Figure 1). Meanwhile, three former staff members evacuated Gaza for safety reasons. Additionally, one was abducted and detained in an Israeli prison, another was severely injured in an airstrike and is now receiving treatment abroad, and a cancer patient was evacuated for treatment. Furthermore, an incoming employee was recently diagnosed with cancer but was denied medical evacuation.

As of November 2024, Al-Aqsa Hospital has treated thousands of orthopedic injuries, a substantial portion of which were directly war-related and required hospitalization. However, the exact numbers are yet to be known and may never be fully documented. Additionally, system gaps have led to underreporting, with cases going unrecorded due to network outages, equipment failures, mass casualty incidents where patients were transferred to other facilities, or hospital evacuations (16). One of the most catastrophic events, the hospital and orthopedic department handled, occurred during the Al-Nuseirat Camp hostage release operation on June 8, which resulted in approximately 1,000 casualties within a matter of hours, dead or injured (17). This included about 100 serious orthopedic injuries, amputations, and open fractures, and many less severe injuries. This single incident is a glimpse of how catastrophic things are and highlights the challenges of the hospital and orthopedic department in providing care in this extraordinary situation.

The orthopedic department’s workload reflects the nature of the injuries sustained in this war, ranging from open fractures, amputations, and crush injuries to shrapnel wounds and degloving injuries (18–22). The injuries were caused by a variety of weapons, including airstrikes, drone attacks, tank missiles, and gunfire (23). Among these, orthopedic trauma cases have been particularly severe, presenting unique challenges in treatment and rehabilitation (24).

## 3 Department workflow and restructure

The orthopedic department at Al-Aqsa Martyrs’ Hospital has undergone significant adaptations to manage the overwhelming influx of patients during the ongoing conflict. These measures were necessary to ensure the efficient delivery of care under extreme resource constraints.

### 3.1 Shift system and staff coordination

**Figure 2:**
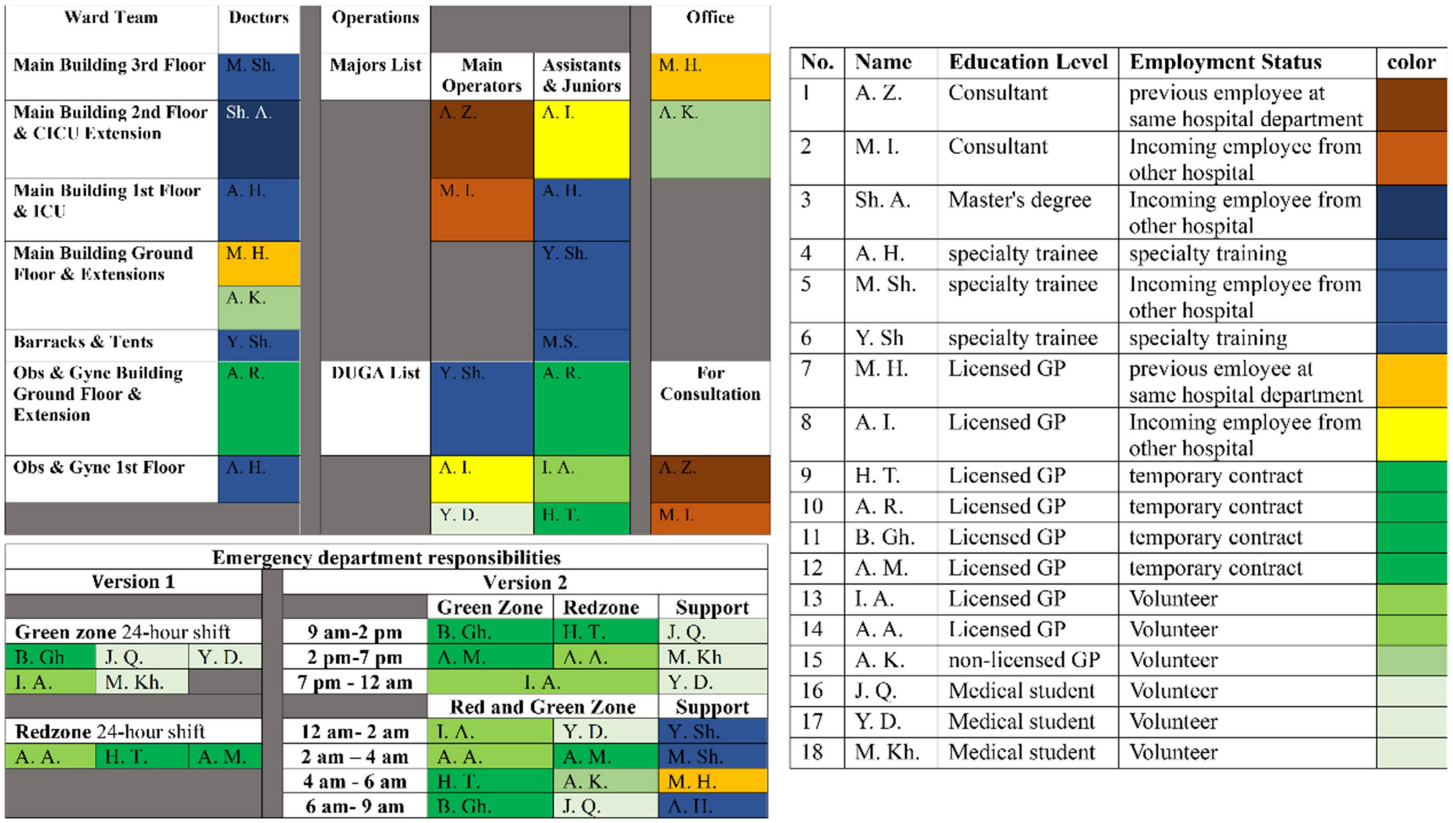
As of November 2024, the orthopedic department operates with three teams on 24 h on followed by 48 h off. This schedule is for one of the three teams at that time. The schedule shows the 18 members of the team color-coded based on their education/experience level and employment status. It then explains the spot each member filled in the team workflow at either emergency, ward, operations, or office responsibilities.

Initially, the department operated on a 24-h shift cycle, which was months later changed to a three-team rotation (24 h on duty, 48 h off) to reduce staff fatigue and ensure continuous patient care. Each team divides responsibilities among members based on skills, training levels, and preferences. This system allows for flexibility, with each team managing its duties in slightly different ways to create a care cycle that ensures the best possible patient care. For instance, Team B organizes its responsibilities in a structured manner to accommodate both urgent and routine tasks effectively as in Figure 2.

### 3.2 Emergency department (ED)

The Emergency Department (ED) at Al-Aqsa is small with 12 beds in the main hall, 5 in the receiving area, and 2 ICU beds. To handle mass casualties, the hospital expanded its emergency capacity with temporary floor extensions and external tents (25). Triage is fluidic, with orthopedic cases classified as either green (non-urgent) or red (urgent).

- Green cases: isolated extremity injuries or stable polytrauma patients with extremity injuries without neurovascular injury, and do not require extensive wound care. These cases are managed in a separate orthopedic room equipped with casting and splinting materials.
- Red cases: injuries may include open fractures, amputations, deep wounds, degloving injuries, retained shrapnel, or unstable polytrauma patients with any extremity injury. These cases are treated in the main ED hall or ICU and require coordination with multiple specialties and sometimes improvising in life-saving interventions.

With approximately 300 daily orthopedic cases, managing these injuries demands significant resilience, stress management, and emotional support from the staff. Effective communication between doctors, patients, and their families is crucial, especially in mass casualties, deaths, and amputation decisions. Doctors assigned to the ED maintain patient lists, summarize diagnoses and priorities, and hand these over to the shift senior at midnight. They are also responsible for continuously notifying and updating seniors regarding urgent cases and coordinating patient transfers to the operating theater or other facilities, ensuring all necessary paperwork is completed.

### 3.3 Inpatient care and follow-up rounds

To manage the influx of patients, the hospital’s capacity was stretched to over three times its intended limit. In addition to the two-floor building, two additional tents and temporary barracks were also set up. But overcrowding remains an issue, many slept for days in corridors. Additionally, patient housing projects were implemented in collaboration with the Palestinian Ministry of Health and international NGOs (INGOs). These included converting two schools into field hospitals and establishing a camp near the hospital to accommodate patients with low care needs, such as physiotherapy and simple wound management. However, these projects were short-lived due to safety concerns, including bombardments and evacuations (26). Furthermore, the hospital has collaborated with INGOs field hospitals, such as Al-Zawayda Hospital, which is run by Médecins Sans Frontières (MSF) (27). Selected cases are referred to these facilities based on pre-set criteria and the hospital’s capabilities, ensuring that patient care is maintained while alleviating pressure on the main hospital.

Orthopedic patients were scattered across different floors, buildings, and extensions, with minimal privacy. Doctors responsible for follow-up manage up to 30 patients each over the 24-h shifts, though this number can exceed a total of 180 during peak times, necessitating six doctors or more. During follow-up rounds, doctors check patient stability, CK (for rhabdomyolysis), and wound care. They also double-check their scheduled surgeries, medications, and their diagnosis as a whole. It is not uncommon for some diagnoses to be missed during mass casualties or for patients to be admitted to the other departments’ responsibilities without proper consultation in the ED. A daily list of patients requiring surgery or wound dressings is compiled and also handed to the shift senior to be merged for the next day’s schedule. Consultations with other specialists or paramedics, like psychologists or physiotherapists, may be required.

### 3.4 Surgical interventions

Before the war, Al-Aqsa Hospital had limited surgical capacity, with orthopedic surgeries scheduled only twice a week. However, the war has led to a massive backlog of surgeries as trauma cases take precedence. According to internal hospital reports, orthopedic surgeries constituted two-thirds of all operations at the hospital for most of wartime. Coordination between the ED and operating room (OR) is crucial, particularly for urgent cases. Common procedures include external fixation (ex-fix), wound dressing, tendon repairs, and amputations. More complex surgeries, such as internal fixation procedures, bone grafts, and limb salvage procedures, are performed when resources allow and have become more common as a definitive management of previously done primary intervention. Minor procedures such as external fixation removal, shrapnel, and bullet removal are frequently done in the ED or inpatient department with potent analgesics.

Most surgeries are conducted under general anesthesia, though nerve blocks are occasionally used depending on the patient’s condition and anesthesia expertise. The department remained overwhelmed with frequent delays of non-urgent surgeries and an ever-growing waiting list, resulting in complications such as non-union, compartment syndrome, and sometimes poor outcomes. Open fractures were often delayed for a few days and treated with Back slab casts over dressings before undergoing surgery, against well-known protocols and guidelines. Same for neck of femur fractures, for example, raising risks for necrosis and the need for later Hip replacement procedures. New urgent admissions often further delay pre-planned surgeries, causing communication issues with patients and their families. Those delayed procedures are also handled to shift seniors for merging with the next day’s schedule. At peak times, there were instances where about 20–30 operations, along with an extensive list of wound dressing, were conducted over the 24-h shift non-stop.

### 3.5 Wound care management

Explosive injuries have led to a variety of wounds, from superficial lacerations to deep, large, or crushing wounds never seen before. Without proper management, these wounds risk developing infections, sepsis, or maggot infestations, with septic wounds often leading to amputations if left untreated (28–30). Wound care is managed according to severity. Initial treatment involves debridement, shrapnel removal, and thorough washing in the ED. Shrapnel removal should always adhere to the “do no harm” protocol to avoid any iatrogenic injuries to neurovascular bundles or tendons, but still were seen in rare occasions.

Detailed documentation is essential to ensure proper follow-up care. This guides decisions during discussions with the shift senior on where to assign patients for their wound care scheduling. Wound care responsibilities were divided and re-organized many times during the war. The Latest update was as follows: minor wounds in the wound care tent for minor wounds, the MSF dressing tent for close follow-up or debridement, or under sedation in the operating room (OR). Patients were usually assigned to their wound care sessions depending on the case and discussion.

**Figure 3:**
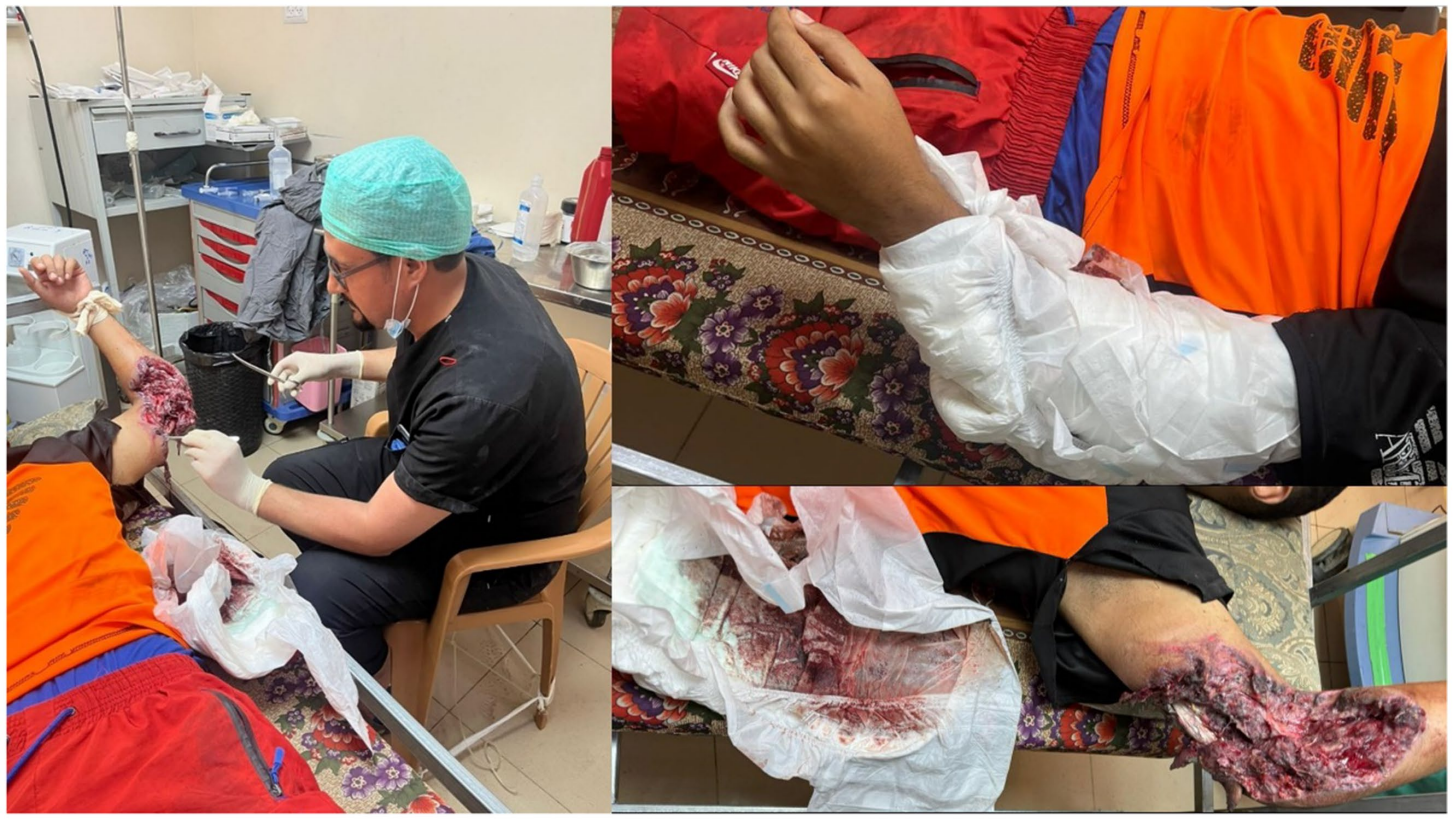
This picture shows the settings and type of wound managed with the DUGA system. It also shows the use of a diaper as a dressing due to the lack of large-size gauze (the picture was taken with permission from both the patient and doctor to be used for academic documentation and publication).

The development of the Dressing Under General Anesthesia (DUGA) operation system has been pivotal for wound management, offering sufficient facilities outside the main operating rooms. One to two of the repurposed previously delivery rooms were used as a theater. This system had its own list similar to major operations but with much more cases, usually 30 cases per day. This system handles more severe cases with large wounds, exposed bones, and raw areas that are highly painful and require advanced and aggressive wound care. This started randomly in corridors with Ketamine and oxygen support, but later progressed to a polished operation system similar to the main operations (see Figure 3).

### 3.6 International collaboration

Collaboration with international missions and NGOs has been vital throughout the conflict. Médecins Sans Frontières (MSF) played a key role in managing orthopedic cases, particularly in wound care. The DUGA system was primarily coordinated and supported by MSF, ensuring optimal wound management for severe cases. Additionally, field hospitals affiliated with the International Medical Corps (IMC), UK-MED, International Committee of the Red Cross (ICRC), and MSF have been instrumental in receiving patients directly from the emergency department during mass casualty events (27, 31–33).

Visiting international teams have also contributed significantly in areas where local expertise was scarce, such as peripheral nerve injury repairs, spine fixation, hand surgery, and bone transplantation (34–36). These teams brought not only their specialized skills but also essential supplies for orthopedic surgeries, including plates, external fixation devices, and gowns, which were crucial to maintaining surgical capacity under extreme conditions. Beyond their clinical contributions, international teams have fostered a sense of solidarity, providing much-needed human support during the conflict.

## 4 A day in the life of the orthopedic department during the war

The orthopedic department operates amidst extraordinary pressures, reflecting the resilience and dedication of its staff. Each day unfolds in a structured yet unpredictable manner, shaped by the demands of wartime medical care.

The day begins at 9 a.m., with staff arriving between 8:30 and 9:30 a.m., many utilizing hospital-provided transportation. However, some staff members, especially those from high-risk areas or overcrowded camps, commute by alternative means, such as donkey or horse carts (37). Others arrive later in the afternoon, balancing their hospital duties with commitments to NGOs that help sustain their financial needs.

The morning routine starts with reviewing the patient handover list and prioritizing high-risk cases, such as new emergencies, polytrauma patients requiring close monitoring, or those in need of urgent surgical intervention. Specialists, who are often updated via messaging platforms like WhatsApp, prepare for operations, including Dressing Under General Anesthesia (DUGA) procedures, and begin as soon as they arrive at the hospital. The operating theater maintains a streamlined process, efficiently rotating patients to minimize delays. Meanwhile, the ward and emergency teams manage patient care, creating a bustling, collaborative environment like a beehive.

In the green zone, doctors and medical students handle patients efficiently with simultaneous examinations, while casting and imaging requests are managed in a seamless flow, assisted by a crowd controller. In the red zone, the focus shifts to severe trauma, with doctors conducting thorough extremity assessments and collaborating with other specialists as needed. As the day progresses, staff take short breaks for prayers and makeshift meals, often relying on canned goods due to the limited availability of hospital provisions (38, 39).

However, this relative calm is frequently disrupted as in the scenario. At 4:15 p.m., an airstrike targets a two-story residential building without prior notice. Civil defense or in-hospital news reporters notify the ED, triggering the deployment of ambulances. The emergency team swiftly mobilizes, reallocating resources and preparing for incoming casualties. At 4:40 p.m., the first victims arrive in civilian vehicles, followed by waves of ambulances and other access vehicles like pickups and carts. Patients include those with mangled limbs and open fractures, along with many others lying on the ER hall floor. Doctors prioritize cases based on severity, age, and polytrauma status, beginning management and going one by one. Critical cases are directed to the ICU, and baseline CBC is carried out for almost all patients with the help of sample transfer with messengers who also help with moving patients around to the imaging rooms and triage areas.

Depending on resource availability, hospital capacity, and prior knowledge of transfer options, cases that need advanced care or operations are triaged through either coordination with the operation theatre team or the referral focal point. For example, in this scenario, one patient requiring an amputation and laparotomy was admitted immediately to the hospital’s operating theater, while another with an open fracture awaited their turn. The other amputation met the transfer criteria to Al-Zawadya MSF field hospital, and two open fractures met the transfer criteria to the IMC field hospital. Our focal point will coordinate with transfer focal points at those hospitals and send injury descriptions and imaging before confirmation. The Redzone team continues to care, conducting mini-procedures such as shrapnel removal, finger stump treatments, and arteriolar ligations, all while adhering to sound anatomical principles.

Amid ongoing care, a second incident—a drone attack on a market at 7 p.m.—introduced additional cases. While vascular consultations were requested, peripheral neurosurgery expertise was unavailable. A similar approach was used for this incident also. Despite the relentless pace, the operation team continued balancing urgent and scheduled surgeries, while ward and office teams maintained their responsibilities. Office team, usually shift senior and another doctor, handle paperwork such as referral forms, discharge notes, lost files, manage consultation from different departments through calls, follow-up with logistical support such as food, internet, and equipment’s shortage, and handle general patient communication inquires of delayed surgeries, pain management, breaking bad news and much more.

By 10 p.m., as the operation team neared the end of their list, the team briefly gathered for dinner, only to be alerted to another potential influx of casualties from the bombing of five residential buildings. Red zone doctors, joined by others from the wards, mobilized to the ER. However, the number of injuries was low, with most individuals in the targeted area either deceased or trapped under rubble. This allowed the team to resume their cold meal. Throughout the night, intermittent emergencies required immediate attention from wards, including re-dressing bleeding wounds of post-operative cases and stabilizing newly admitted trauma patients. By midnight, the next day’s surgical schedule was finalized for both major operations and DUGA procedures, reflecting meticulous planning despite overwhelming demands.

The night often extended into the early hours in a similar pattern. At 7 a.m., a new wave of casualties from a tank missile strike arrived, forcing the red zone team to miss their buses and remain on duty. Among the injured were a mother and her 3-year-old daughter, both requiring urgent upper limb amputations, and the father, who sustained an open femur fracture. These cases were communicated to the operating theater team for prioritization on the surgical list.

As the day concluded, the orthopedic team managed over 300 ED and 85 ward cases, performed nine major operations, and completed 35 DUGA procedures. These accomplishments underscored their dedication to preserving life and functionality. The team’s collective efforts demonstrate remarkable resilience, navigating the physical and psychological toll of war while maintaining a high standard of care. Each day is a testament to their unwavering commitment to preserving life under the most challenging circumstances.

## 5 Healthcare system resilience: challenges and policy implications

The strategies implemented by the orthopedic department at Al-Aqsa Martyrs’ Hospital have been critical in providing care to war-injured patients. However, the department continues to confront significant challenges, reflective of deeper systemic issues within Gaza’s healthcare system, exacerbated by the conflict. These challenges highlight the urgent need for policy interventions to enhance the resilience and functionality of healthcare services during prolonged crises.

### 5.1 Infrastructure and resources

The hospital, including the orthopedic department, faces severe shortages of beds and medical supplies, such as orthopedic padding, which have been further strained by the growing patient population (9). The influx has overcrowded the hospital, forcing patients into corridors (40). This has also compromised patient supervision and left little space for new admissions. Additionally, the hospital’s limited number of operating rooms and critical shortages in orthopedic surgical tools, such as bone loss devices, have further delayed surgeries, negatively impacting patient outcomes.

To address these challenges, resource management must improve through:

- Pre-positioning essential medical supplies to ensure continuous care for extended periods, especially during times of blockade.
- Strengthening supply chains with the support of international organizations to protect against theft, targeting, and interference (41, 42).
- Implementing flexible and secure resource distribution systems, reinforced by real-time tracking mechanisms to ensure efficient allocation of critical resources.

Flexible and temporary units to accommodate patient overflow should be prioritized as needed. Additionally, organizing inpatient housing is necessary to ensure that those with higher medical needs remain in the hospital while others requiring lower levels of care are directed to field facilities for post-operative care or physiotherapy. Regular patient assessments of their care level should be done to optimize the care cycle.

### 5.2 Emergency response

The department’s struggle to manage mass casualty events reflects broader issues in the hospital’s emergency preparedness and the intensity of bombardments with the overload the department faces. To enhance emergency response and surge capacity, policies should focus on:

- Clear triage protocols: develop orthopedic-specific emergency triage systems that prioritize critical cases such as limb-threatening injuries while ensuring non-emergency cases are also maintained.
- Emergency referral systems: create pathways to divert mass casualties to nearby field hospitals for complex orthopedic cases, such as limb salvage surgeries, which take a lot of time and effort.
- Crisis-specific training: train orthopedic staff and volunteers in orthopedic trauma management, including advanced wound care, along with problem-solving skills for care in resource-limited settings.

### 5.3 Clinical management

The orthopedic department faces clinical management challenges due to the lack of pain management protocols, delays in wound care, and the absence of a proper outpatient clinic. Without standardized protocols for wound care, tetanus vaccination, and antibiotic usage, treatments are inconsistent, leading to complications like infections and sepsis.

To improve clinical management, policies should include:

- Standardized protocols: introducing uniform protocols for debridement, antibiotic usage, and post-operative wound monitoring can reduce the risk of sepsis and improve recovery rates.
- Pain management center: establishing a dedicated pain management framework for both acute and chronic cases, tailored to orthopedic trauma patients, will enhance patient comfort and reduce reliance on long-term opioids.
- Outpatient clinic activation: reinstating an outpatient orthopedic clinic will enable better post-operative care, reduce ED green zone congestion, and improve long-term outcomes for patients with complex injuries.

### 5.4 Operational coordination

Communication gaps between medical, paramedical, and other departments have hindered the timely delivery of care. Furthermore, inadequate coordination of non-medical services, such as cleaning and catering, has exacerbated operational inefficiencies. To address these issues, policies should focus on:

- Interdisciplinary collaboration: regular case reviews involving surgeons, physiotherapists, and psychologists can ensure comprehensive patient management.
- Support services: improved coordination with non-medical teams, such as IT and cleaning units, can minimize delays and effort lost on low-priority tasks.

### 5.5 Staff wellbeing and volunteer engagement

The orthopedic department’s staff face immense psychological burdens, compounded by a lack of recognition and mental health support. Continuous professional development and support programs are essential to improving staff morale and retention. Key recommendations include:

- Mental health support: providing access to counseling and peer support groups can mitigate burnout and psychological trauma.
- Professional development: offering training opportunities in advanced orthopedic techniques and orthopedic trauma management will improve staff capacity to handle complex trauma cases.
- Recognition programs: establishing structured onboarding and recognition programs for medical students and volunteers will enhance their contributions while fostering a sense of purpose, boosting morale, and enhancing retention.

### 5.6 International collaboration

International NGOs have played a key role in supporting the orthopedic department through either hospital-level collaboration or medical delegations of professionals. However, poor coordination between local healthcare authorities and international teams has led to the misallocation of resources and some duplication of efforts. Establishing centralized coordination bodies will better integrate international aid with local healthcare needs, ensuring that external resources are used effectively and align with local priorities. These partnerships have the potential to lead to long-term collaborations, which could further strengthen Gaza’s healthcare system. However, the reliance on international NGOs must be balanced with policies that promote local sustainability, including training programs for local staff to improve skills and capacity.

### 5.7 Long-term healthcare resilience

To build long-term resilience in Gaza’s healthcare system, policies must address both immediate and future needs. Key recommendations include:

- Infrastructure investment: rebuilding damaged hospitals and expanding current hospitals’ operation theatres and inpatient capacity can create adaptable healthcare facilities capable of functioning under extreme conditions. For example, resuming the operation theaters reconstruction at Al-Aqsa Hospital along with the expansion of another building dedicated to long-term orthopedic and rehabilitation care of the injured.
- Rehabilitation capacity: the Ministry of Health and other partners must establish a comprehensive rehabilitation program for the increasing number of patients with amputations and war-related disabilities. This includes creating a patient registry and ensuring early integration of physiotherapy and psychological support. Orthotics, prosthetics, and artificial limb services—including production, importation, and fitting—must be urgently scaled up to ensure earlier recovery and social reintegration.
- National emergency response plan: develop a national plan that integrates local hospitals, governmental authorities represented by Ministry of Health, and international organizations to ensure a cohesive and effective response to this and future crises in different health aspects, including orthopedics.
- Research and advocacy: conducting research on wartime orthopedic care and publishing findings can attract international attention and support for the department’s needs.

By addressing these targeted challenges, the orthopedic department at Al-Aqsa Martyrs’ Hospital can not only improve immediate patient care but also build a foundation for long-term resilience in the face of ongoing conflict.

## 6 Methodological constraints

This study employs a retrospective case study design to analyze the adaptation of orthopedic care at Al-Aqsa Martyrs’ Hospital during the ongoing war in Gaza and the challenges the department has faced. Data sources include Internal hospital records, direct observation, staff experiences, and informal discussions with orthopedic department representatives at Al-Aqsa Martyrs’ Hospital. The data used here represents the period from October 2023 to November 2024. Triagulation and double verification of findings and notes were made whenever possible. There was no identifying data. Due to wartime conditions, formal institutional review board (IRB) approval was not feasible; however, all observations were conducted with respect for patient confidentiality and professional ethics and under emergency considerations laid out in the Declaration of Helsinki, and in accordance with the guidelines of the Ministry of Health. Efforts were made to ensure comprehensive coverage of the efforts at the orthopedic department despite the chaotic nature of war. This case has also been written based on information as of November 2024. However, the hospital is trying to improve things, so things might change.

## Data Availability

The original contributions presented in the study are included in the article further, inquiries can be directed to the corresponding author.
